# Striatal Neuroinflammation Promotes Parkinsonism in Rats

**DOI:** 10.1371/journal.pone.0005482

**Published:** 2009-05-08

**Authors:** Dong-Young Choi, Mei Liu, Randy L. Hunter, Wayne A. Cass, Jignesh D. Pandya, Patrick G. Sullivan, Eun-Joo Shin, Hyoung-Chun Kim, Don M. Gash, Guoying Bing

**Affiliations:** 1 Department of Anatomy and Neurobiology, University of Kentucky, Lexington, Kentucky, United States of America; 2 Spinal Cord and Brain Injury Research Center, University of Kentucky, Lexington, Kentucky, United States of America; 3 Neuropsychopharmacology and Toxicology Program, College of Pharmacy, Kangwon National University, Chunchon, South Korea; University of Nebraska, United States of America

## Abstract

**Background:**

Sporadic Parkinson's disease (PD) is a progressive neurodegenerative disorder with unknown cause, but it has been suggested that neuroinflammation may play a role in pathogenesis of the disease. Neuroinflammatory component in process of PD neurodegeneration was proposed by postmortem, epidemiological and animal model studies. However, it remains unclear how neuroinflammatory factors contribute to dopaminergic neuronal death in PD.

**Findings:**

In this study, we analyzed the relationship among inducible nitric oxide synthase (iNOS)-derived NO, mitochondrial dysfunction and dopaminergic neurodegeneration to examine the possibility that microglial neuroinflammation may induce dopaminergic neuronal loss in the substantia nigra. Unilateral injection of lipopolysaccharide (LPS) into the striatum of rat was followed by immunocytochemical, histological, neurochemical and biochemical analyses. In addition, behavioral assessments including cylinder test and amphetamine-induced rotational behavior test were employed to validate ipsilateral damage to the dopamine nigrostriatal pathway. LPS injection caused progressive degeneration of the dopamine nigrostriatal system, which was accompanied by motor impairments including asymmetric usage of forelimbs and amphetamine-induced turning behavior in animals. Interestingly, some of the remaining nigral dopaminergic neurons had intracytoplasmic accumulation of α-synuclein and ubiquitin. Furthermore, defect in the mitochondrial respiratory chain, and extensive S-nitrosylation/nitration of mitochondrial complex I were detected prior to the dopaminergic neuronal loss. The mitochondrial injury was prevented by treatment with L-N^6^-(l-iminoethyl)-lysine, an iNOS inhibitor, suggesting that iNOS-derived NO is associated with the mitochondrial impairment.

**Conclusions:**

These results implicate neuroinflammation-induced S-nitrosylation/nitration of mitochondrial complex I in mitochondrial malfunction and subsequent degeneration of the nigral dopamine neurons.

## Introduction

Microglial activation is a pathological hallmark of neurodegenerative diseases including Parkinson's disease (PD) [1]. Microgliosis is a normal response in the damaged CNS, which can promote sprouting of injured neurons by providing neurotrophic factors [2]. On the other hand, the activated microglia may be destructive to neurons by releasing inflammatory molecules such as nitric oxide (NO), and cytokines [3], [4]. While the role of activated microglia in the parkinsonian brain is controversial, the observation of persistent microgliosis in the substantia nigra of parkinsonian patients, and 1-methyl-4-phenyl-1,2,3,6-tetrahydropyridine-exposed humans and animals has led to a postulation that the chronic inflammatory response might contribute to loss of the dopamine neurons [5]–[8]. A study using positron emission tomography with [^11^C](*R*)-PK11195, a radiotracer for activated microglia and [^11^C]CFT, a dopamine transporter marker reported that microglial activation level in the midbrain is positively correlated with severity of motor impairment and dopaminergic terminal loss in the putamen of early PD patients, suggesting microglial neuroinflammatory response may be related to progressive neurodegeneration of the disease [9]. In line with the observation, an epidemiological study illustrated that the risk of developing PD is significantly reduced by regular use of non-steroidal anti-inflammatory drugs such as ibuprofen [10]. In addition, reactive microglia express increased levels of inflammatory enzymes including inducible nitric oxide synthase (iNOS) and cyclooxygenase-2 in the substantia nigra of PD brain [11]. Importantly, these enzymes appear to play a pivotal role in the loss of dopaminergic neurons in animal models of PD [12]–[14].

iNOS is expressed in microglia during neuroinflammation and produces excessive amount of NO, which can cause death of neurons [15]. The gaseous molecule released at extramitochondrial sites can diffuse into the mitochondria and react with mitochondria-derived superoxide anion to form peroxynitrite, which accounts for much of the disruption of mitochondrial function [16]. Moreover, peroxynitrite inhibits the activity of manganese superoxide dismutase, which could enhance formation of superoxide anion and peroxynitrite [17]. It has been documented that NO and its metabolite peroxynitrite inhibit mitochondrial respiration via S-nitrosylation and nitration of complex I, respectively [18]. Impairment in the mitochondrial electron transport chain has been suggested to be a critical factor in the pathogenesis of PD, as defect in mitochondrial complex I is consistently detected in PD patients [19], [20] and mitochondrial toxins induces parkinsonism [21]–[23]. The dopaminergic neurons can be particularly susceptible to NO neurotoxicity because dopamine potentiates NO-induced impairment in mitochondrial respiration [24]. Furthermore, metabolism of dopamine by monoamine oxidase, a mitochondrial enzyme may enhance the mitochondrial lesion in the dopamine-producing neurons [25].

We and other groups have demonstrated that intranigral [26]–[28] or intrapalidal [29] lipopolysaccharide (LPS) induces death of the nigral dopaminergic neurons through microglial activation. However, these animal models did not recapitulate some cardinal features of PD such as progressive dopaminergic neurodegeneration, Lewy body-like intracytoplasmic inclusion and parkinsonian behavioral impairments. Furthermore, underlying mechanism(s) by which microglial neuroinflammation kills the nigral dopaminergic neurons has not been fully delineated. This study was designed to demonstrate striatal neuroinflammation-induced dopaminergic neurodegeneration and explore how neuroinflammation mediates the loss of dopamine-producing neurons. Here, we report that LPS-induced striatal inflammation causes the impairment of the mitochondrial respiratory chain in both the substantia nigra and striatum, which was followed by progressive degeneration of the dopamine nigrostriatal pathway, behavioral impairment, and accumulation of α-synuclein and ubiquitin in the remaining nigral dopaminergic neurons. In addition, our results indicate that NO produced by iNOS is related to S-nitrosylation/nitration of mitochondrial complex I which might lead to mitochondrial dysfunction and the subsequent dopaminergic neurodegeneration.

## Methods

### Animals and surgery

All animal experiments were performed according to the NIH Guide for the *Care and Use of Laboratory Animals* and were approved by the University of Kentucky Institutional Animal Care and Use Committee. 3 month-old male Sprague-Dawley rats (Harlan, Indianapolis, IN) were housed under a twelve hour light-dark cycle with free access to food and water in the Division of Lab Animal Resources at the University of Kentucky. For the injection of LPS (*Salmonella minnesota*; Sigma-Aldrich, St Louis, MO), rats were deeply anesthetized with sodium pentobarbital (50 mg/kg i.p.) and were positioned in a stereotaxic frame (Stoelting Co., Wood Dale, IL) with the incisor bar at the level of the ear. LPS dissolved in saline (2.5 µg/µl) was injected into the right striatum (3 µl/site) using the following coordinates (in mm): site 1, anteroposterior (AP) 1.0, mediolateral (ML) 2.0, dorsoventral (DV) −5.5; site 2, AP 1.0, ML 3.5, DV −6.0; site 3, AP −0.5, ML 2.5, DV −5.0; site 4, AP −0.5, ML 4.0, DV −6.5. Saline was injected into the left striatum with parallel coordinates. Placebo animals received saline injections into the right and left striatum with the same regimen. After surgery, animals were kept on heating pad until recovery from surgery and subcutaneous saline was given for aid in post-operative recovery. In order to investigate the detrimental effect of iNOS on the mitochondria, animals were treated with L-N^6^-(l-iminoethyl)-lysine (L-NIL, 5 mg/ml/kg, i.p., Cayman Chemical, Ann Arbor, MI) or its vehicle (sterilized saline) 20 min before, and one day and two days after LPS injection.

### Histopathology

Animals were perfused with 4% paraformaldehyde three days, one week, two weeks, and four weeks after LPS injections (n = 5–6/group) for immunohistochemistry and histopathological examinations. Coronal sections (30 µm) were cut through the entire brain using a sliding microtome. Immunohistochemistry, with slight modifications, was performed as previously described [28]. Briefly, every sixth section from the region containing the striatum or substantia nigra was blocked with 5% bovine serum albumin for 1 hr at room temperature. The brain sections were immunolabeled with antibody to tyrosine hydroxylase (TH, Calbiochem, San Diego, CA, 1∶3,000), MHC class II (OX-6, Serotec, Raleigh, NC, 1∶1,000) or dopamine- and cAMP-regulated phosphoprotein polypeptide (DARPP-32, Chemicon, Temecula, CA, 1∶1,000, 24 hrs at 4°C). After multiple washings, sections were incubated with species-appropriate secondary antibody (1∶1,000; Vector Laboratories, Burlingame, CA). The final antigen-antibody complex was visualized using an Avidin-biotin complex (ABC kit, Vector laboratories) method and 3,3′-diaminobenzidine tetrachloride as a chromagen (Sigma-Aldrich).

Colocalization of TH with α-synuclein, or ubiquitin was assessed as previously described [29]. Briefly, substantia nigra-containing sections were incubated with primary polyclonal antibody against α-synuclein (1∶500; Sigma-Aldrich) or ubiquitin (1∶1,000; Sigma-Aldrich) overnight at 4°C. The sections were incubated in Alexa Fluor 488 goat anti-rabbit secondary antibody (1∶1,000; Molecular Probes Inc., Eugene, OR) for 1 hr, at room temperature. The sections were subsequently incubated with mouse anti-TH primary antibody (1∶1,000; Calbiochem) overnight at 4°C followed by incubation for 1 hr in Alexa Fluor 568 goat anti-mouse IgG secondary antibody (1∶1,000; Molecular Probes Inc.) at room temperature. The fluorescent specimens were first assessed by an Axioplan 2 microscope (Carl Zeiss, Inc.) and images were acquired by using an Axiocam digital camera connected to a computer equipped with Axiovision 3.0 software (Carl Zeiss, Inc.). Fluorescent preparations were also examined using the Leica TCS SP laser scanning confocal imaging system (Leica Microsystems, Inc., Buffalo, NY). Images were viewed on a Leica DM RXE upright microscope. Photomicrographs were captured simultaneously for both fluorophores [Alexa Fluor 488 (green) and Alexa Fluor 568 (red)] by using argon and krypton lasers, respectively. Regions exhibiting colocalization of the red and green emitters produced yellow fluorescence.

Another set of sections were rinsed in PBS and then incubated in PBS containing proteinase K (20 µg/ml) for 30 mins at room temperature. After multiple washings in PBS, these sections were subjected to immunostaining with antibodies to α-synuclein (1∶5,000, Chemicon, Temecula, CA) or ubiquitin (1∶5,000, Sigma-Aldrich).

Standard Nissl staining was performed to assess neuronal morphology and population in the substantia nigra. Degenerative neuronal cell bodies and neurites in the substantia nigra and striatum were visualized using the FD Neurosilver kit (FD NeuroTechnologies, Ellicott City, MD), which was performed according to the manufacturer's protocol. Photomicrographs were taken by using an Axiocam digital camera connected to a computer equipped with Axiovision 3.0 software (Carl Zeiss, Inc., Thornwood, NY).

### Quantification of immunohistochemistry

The number of TH-positive neurons in the substantia nigra was determined using the computerized optical fractionator method of the Bioquant system (Memphis, TN, USA), which was described previously [30], [31]. Striatal TH-positive fiber density was measured as previously described [32]. Briefly, striatum-containing sections were picked at seven rostrocaudal levels, covering the entire head and tail of the striatum, corresponding to: (i) AP +1.60; (ii) AP +1.00; (iii) AP+0.20; (iv) AP −0.30; (v) AP −0.90; (vi) AP −1.40 and (vii) AP −2.10 relative to bregma. TH-positive fiber density was measured by using Bioquant Image Analysis software. Each value was corrected for non-specific background by subtracting the optical density of corpus callosum.

### HPLC analysis for levels of striatal neurochemicals

Animals were killed four weeks following LPS challenge for determination of striatal dopamine, serotonin and their metabolites levels (n = 7/group). Levels of striatal dopamine, 3,4-dihydroxyphenylacetic acid (DOPAC), homovanillic acid (HVA), serotonin, and 5-hydroxyindole acetic acid (5-HIAA) were determined by using HPLC. Analyses were performed as previously described [33]. Briefly, tissue samples were sonicated in 300 µl of cold 0.1 M perchloric acid containing dihydroxybenzylamine as an internal standard. The supernatant was separated by centrifugation at 12,000 *g* for five minutes, and was transferred to Millipore Ultrafree centrifugal filters (pore size, 0.22 µm), and then were spun at 12,000 *g* for one minute. The filtrate was diluted with HPLC mobile phase, and 50 µl was injected onto the HPLC column. The HPLC system consisted of a Beckman Model 507 autoinjector, a Beckman Model 118 pump, and an ESA Model 5200A Coulochem II electrochemical detector with a Model 5011 dual-detector analytical cell (detector 1 set at +350 mV and detector 2 set at −300 mV). An ESA Hypersil ODS 3 µm particle C_18_ column (80×4.6 mm) was used for separations. Flow rate was 1.4 ml/min and the mobile phase was a pH 4.1, 0.17 M citrate–acetate buffer (containing 5 mg/l EDTA, 70–80 mg/l octanesulfonic acid, and 7–8% methanol). Chromatograms were recorded from both detectors using two dual-channel strip chart recorders. Retention times of standards were used to identify peaks, and peak heights were used to calculate recovery of internal standard and amount of dopamine and metabolites. Tissue monoamine concentrations of dopamine, DOPAC, HVA, serotonin, and 5-HIAA are expressed as ng/mg wet tissue.

### Behavioral assessment

Test for amphetamine-induced rotational behavior was performed four weeks following LPS injection (n = 5–6/group). For the test, animals were placed in a hemispherical bowl immediately after receiving 5 mg/kg amphetamine injection (i.p.). The behavior of each animal was monitored through an automated video-tracking system, and the number of ipsilateral 360° turns was determined for 90 minutes per animal.

Cylinder test is a motor assessment of forelimb asymmetry, and was performed as described previously with slight modifications [34]. Briefly, rats were individually put into a glass cylinder (20 cm diameter, 34 cm height) and were video recorded until they touched the cylinder wall with their forelimbs 20 times. The recordings were analyzed by an investigator who was not aware of the identity of the rats. The data are presented as the asymmetric score calculated by the following formula: (Right touch−Left touch)/(Right touch+Left touch+Both touch)(n = 5–6/group).

### Mitochondrial isolation and measurement of their respiration

Mitochondria were isolated using discontinuous Ficoll gradient and differential centrifugation with nitrogen disruption, and respiration was assessed as previously described with slight modifications [35]. Briefly, rats were killed three days after LPS injections and the nigral and striatal tissues were immediately and carefully dissected. Two unilaterally injected striata or four nigra, ipsilateral to the injections, had to be pooled to obtain one sample (n = 6–9/group). The tissues were homogenized with ice-cold isolation buffer (pH 7.2, 215 mM mannitol, 75 mM sucrose, 0.1% bovine serum albumin, 20 mM HEPES, 1 mM EGTA) and the crude mitochondrial fraction was obtained by differential centrifugation and nitrogen disruption. Further purification was performed using a Ficoll gradient and differential centrifugation. Mitochondrial respiratory rates were measured using a Clark-type electrode in a continuously stirred and sealed thermostatically controlled chamber (Oxytherm System, Hansatech Instruments Ltd., King's Lynn, Norfolk, UK) maintained at 37°C. 25 to 40 mg of isolated mitochondrial protein was placed in the chamber containing 250 ml of KCl-based respiration buffer (125 mM KCl, 2 mM MgCl_2_, 2.5 mM KH_2_PO_4_, 0.1% BSA, 20 mM HEPES at pH 7.2) and allowed to equilibrate for 1 min. State II respiration was initiated by the addition of complex I substrates, pyruvate (5 mM) and malate (2.5 mM), to monitor. Then ADP (150 mM) were added to the mitochondria to assess state III respiratory rate for 2 mins, followed by the addition of oligomycin (2 mM) to monitor state IV respiration rate for an additional 2 mins. For the measurement of uncoupled respiratory rate, FCCP (2 mM) was added to the mitochondria in the chamber and oxygen consumption rate was measured for another 2 mins. This was followed by the addition of succinate (10 mM) to monitor complex II-driven respiration. The results were the rates of oxygen consumption in nanoatoms of oxygen/min/mg protein and presented as percentage of control.

### Assessment of mitochondrial protein S-nitrosylation/nitration

Approximately, 200 µg of mitochondrial protein was solubilized in RIPA buffer containing 1% lauryl maltoside (Sigma-Aldrich) and centrifuged at 20,000g for 30 min to collect the supernatant. The mitochondrial solution was incubated at 4°C for 24 hr with 15 µl of agarose beads, irreversibly cross-linked to complex I specific antibodies (MitoSciences, Eugene, OR). The beads were collected by centrifugation and washed three times with PBS containing 0.05% laruryl maltoside. The beads were then resuspended in 50 µl of sample loading buffer with non-reducing condition and agitated for 10 min. After gentle centrifugation, 10 µl of the supernatant was loaded into a 12% SDS-PAGE gel and proteins were resolved. The proteins were transferred to PVDF membrane and detected by incubation with a polyclonal antibody against 3-nitrotyrosine (Upstate Biotechnology, Lake Placid, NY) or S-nitrosylcystein (Sigma-Aldrich), which was followed by horseradish peroxidase labeled goat anti-rabbit IgG (Sigma-Aldrich). Chemiluminescent detection with the ECL Plus kit and exposure to x-ray film was performed, and protein levels were quantified by the Scion Image software (Scion Corporation, Frederick, MD) (n = 4/group).

### Western blot analysis

Animals were sacrificed six hours, one day, and three days after LPS or saline injections. The striatum and substantia nigra were dissected out on ice. Tissues were homogenized in ice-cold lysis buffer and centrifuged (10,600 g) to collect the supernatant. All of the samples were kept at −70°C until they were used for analysis. Next, 15 µg of protein from each sample was aliquoted, and after addition of loading buffer, the protein was loaded and resolved using a 12% SDS-PAGE gel. Protein on the gel was transferred to a nitrocellulose membrane, which was blocked in 5% fat-free milk at 4°C overnight. The membrane was incubated in primary antibodies to iNOS (1∶1,000, Upstate Biotechnology) or DARPP-32 (1∶4,000) at room temperature for one hour. Then, the membrane was rinsed with tris-buffered saline three times for 15 min each before incubation in secondary antibody (goat anti-rabbit, 1∶2,000; Sigma-Aldrich) for one hour. This was followed by treatment with the ECL chemiluminescent reagents (Amersham Biosciences, Piscataway, NJ) and exposure to film. A density measurement for each band was performed with the Scion Image software (Scion Corporation). Background values from an equivalent area near each lane were subtracted from each band to calculate mean band density and iNOS or DARPP-32 immunoreactivity was normalized by density of β-actin bands in the same membrane to correct loading error (n = 3–4/group).

### Statistical analysis

Animals were randomly grouped, and tests of variance homogeneity, normality, and distribution were performed to ensure that the assumptions required for standard parametric analysis of variance (ANOVA) were satisfied. The Systat 10 software (SPSS Inc., Chicago, IL) was used to perform statistical analyses by using the linear correlation unpaired test, Student's *t-*test or ANOVA followed by a protected least significant differences post hoc test only following a positive F test result. Statistical significance was set at p<0.05. The linear correlation unpaired test was performed to analyze correlation between loss of nigral TH-positive cells and time. The ANOVA was used for analysis of the stereological cell counts, mitochondrial respiration data, RNase protection assay, and western blot. The Student's *t*-test was used to analyze HPLC and behavioral data. Data are expressed as means ± s.e.m.

## Results

### 1. Progressive degeneration of the dopamine nigrostriatal system

**Figure 1:**
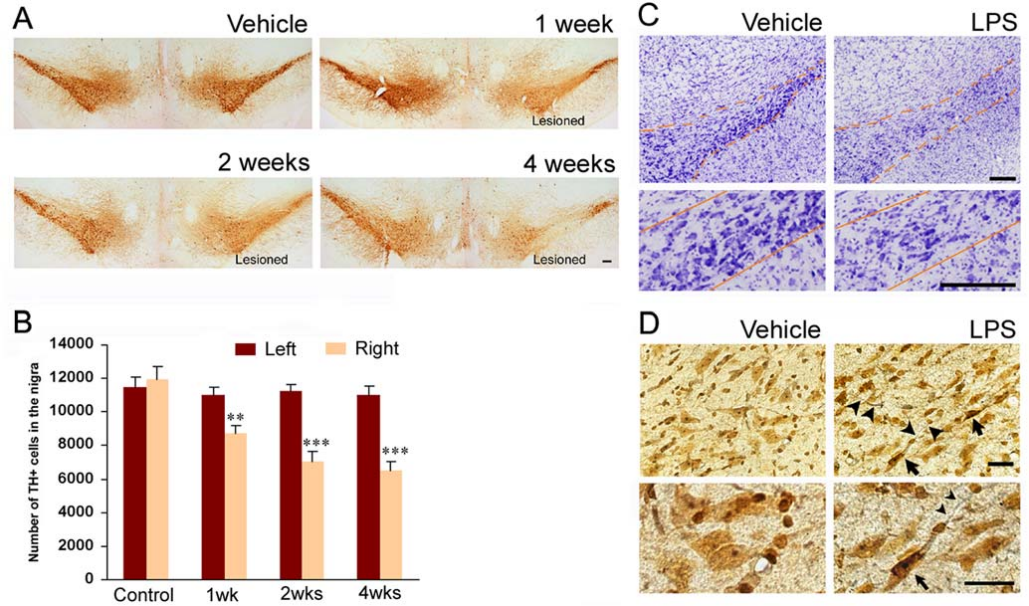
Progressive degeneration of the nigral dopaminergic neurons after intrastriatal LPS. (A) Representative TH immunostaining of coronal midbrain sections demonstrates that the numbers of TH-positive neurons and fibers in the substantia nigra pars compacta are gradually reduced by intrastriatal LPS injection. Note that TH-positive neurons in the medial substantia nigra pars compacta and ventral tegmental area are spared; scale bar: 200 µm. (B) Stereological cell counts of the TH-positive neurons in the substantia nigra pars compacta (n = 5–6/group, ** p<0.01, *** p<0.001). (C) The substantia nigra pars compacta is outlined with an orange dashed line (top). High magnification image of Nissl stainings suggest loss of the nigral dopaminergic neurons, at four weeks following LPS injection (bottom); scale bar: 200 µm. (D) Silver staining is hardly seen in the substantia nigra ipsilateral to vehicle treatment. However, abundant silver grain-deposits are observed in the neurons (arrows) and fibers (arrow heads) in the substantia nigra ipsilateral to the intrastriatal LPS injections, indicating there is ongoing neurodegenerative process in the region. Scale bar: 20 µm.

**Figure 2:**
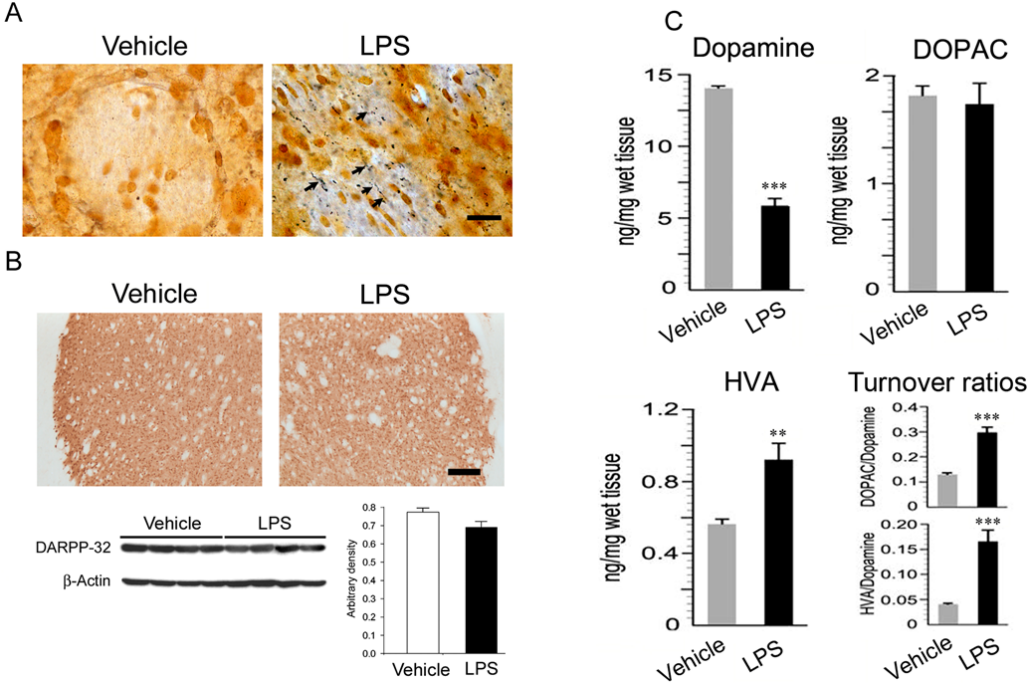
Axonal terminal degeneration in the striatum following intrastriatal LPS. (A) Silver staining reveals that there is no silver-positive stained fibers in the vehicle treated striatum while an abundance of silver grain-deposits are observed in the LPS injected striatum, suggesting the degeneration of axonal fibers (arrows). Scale bar: 20 µm. (B) Immunostaining for DARPP-32 shows that the GABAergic neurons are intact following LPS injections. Western blot for DARPP-32 and its quantification indicate that there is no significant alteration in the expression of DARPP-32 after LPS challenge. Scale bar: 200 µm. (C) HPLC analysis shows that intrastriatal LPS injection depletes 58% of the striatal dopamine relative to control at four weeks. The DOPAC level is not affected; however, HVA is significantly increased. The turnover ratios of DOPAC/dopamine and HVA/dopamine are dramatically increased (n = 7/group; ** p<0.01, *** p<0.001).

To characterize and quantify the loss of dopaminergic neurons in the midbrain after LPS injection, immunostaining with an antibody against TH and non-biased stereological estimation of the TH-positive neurons in the substantia nigra were performed. Abundant TH-positive cell bodies and fibers existed in the substantia nigra and ventral tegmental area of placebo and saline-treated side. In contrast, the number of TH-positive cells and fibers progressively decreased in the substantia nigra ipsilateral to the LPS injection, while TH-positive neurons in the ventral tegmental area were spared (Fig. 1A). Stereological estimation of the spared nigral TH-positive cells showed a progressive and significant loss of the cells: 21% at one week (p = 8×10^−3^), 38% at two weeks (p = 1×10^−5^), and 41% at four weeks (p = 2×10^−7^; Fig. 1B). Stereological analysis indicated that the number of TH-positive cells in the ventral tegmental area was not significantly affected by LPS injection (98.9±2.9% at one week, 100.2±7.1% at two weeks and 97.3±7.4% at four weeks vs. control). Nissl staining of adjacent sections showed fewer large neurons in the substantia nigra, consistent with the loss of dopaminergic neurons four weeks after LPS injection (Fig. 1C). To detect ongoing degenerative events of the nigrostriatal dopaminergic system, silver staining was performed. No nigral degeneration was detected in the substantia nigra ipsilateral to the vehicle-injected striatum (Fig. 1D). However, nigral neurons with silver deposits in their cell bodies or fibers were observed in the substantia nigra ipsilateral to the LPS-injected side (Fig. 1D). In addition to the loss of dopaminergic cell bodies in the substantia nigra, silver staining of striatal sections revealed that axon terminals were undergoing degeneration by LPS-induced inflammation, as shown by dense staining with silver grains (Fig. 2A). Density of striatal TH-positive fiber was also significantly reduced four weeks after LPS injection (Fig. S1, 15%, p = 0.001). In contrast, the immunostaining for DARPP-32 indicated that the population of gamma-aminobutyric acid (GABA) neurons in the striatum did not seem to be affected by the LPS infusions (Fig. 2B, top panel). In agreement with the immunostaining, expression level of DARPP-32 was not significantly altered by LPS challenge as determined by Western blot analysis (Fig. 2B, bottom panel). Consistent with the degeneration of the nigrostriatal system, the level of striatal dopamine significantly declined to 42% of the control level, four weeks after LPS injection (p = 1×10^−9^). The turnover ratio of DOPAC (p = 1×10^−6^) or HVA (p = 1×10^−5^) to dopamine was significantly increased, as occurs in PD [36]. There was also a significant increase in HVA level (p = 0.004) but not DOPAC following LPS (Fig. 2C), suggesting an alteration in dopamine metabolism. Serotonin but not its primary metabolite, 5-HIAA was also significantly decreased (p = 0.0002) leading to a significant increase in the turnover ratio (5-HIAA/serotonin; p = 2×10^−5^) (Fig. S2 **)**, which may indicate that serotonergic neurons were affected by LPS infusion.

### 2. Intracytoplasmic accumulations of α-synuclein and ubiquitin

**Figure 3:**
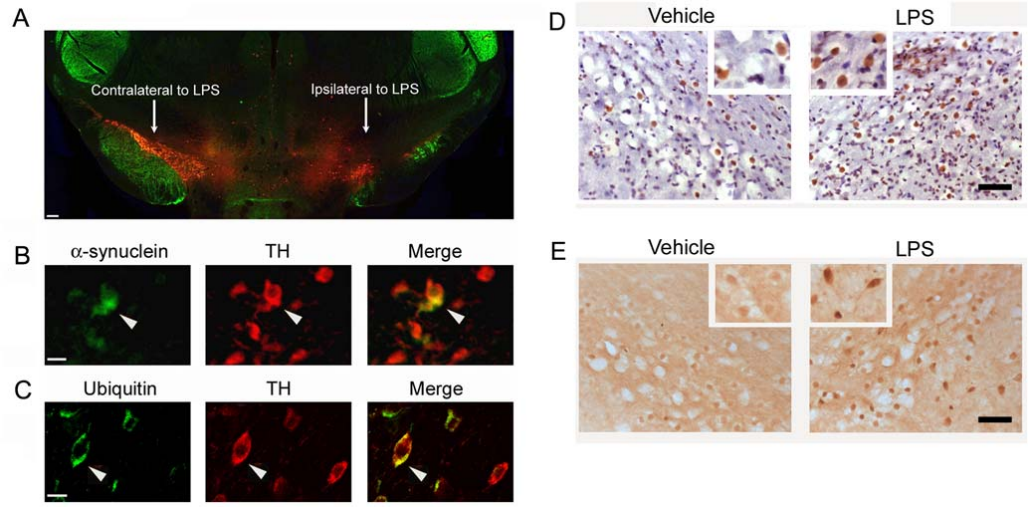
Cytoplasmic accumulation of α-synuclein and ubiquitin in the nigral TH-positive neurons at four weeks after intrastriatal LPS. (A) Photomicrograph of double immunofluorescent labeling with antibodies against TH (red) and α-synuclein (green) show that intrastriatal LPS mediates marked TH-positive cell loss in the substantia nigra ipsilateral to the injection. Scale bar: 100 µm. (B) High magnification images of the top photograph demonstrate that some of the spared TH-positive neurons have accumulated α-synuclein in their cytoplasm (arrow heads). Scale bar: 20 µm. (C) Immunofluorescent staining displays ubiquitin accumulation in the cytoplasm of the nigral TH-positive neurons (arrow heads). Scale bar: 20 µm. (D–E) Increase of proteinase K-resistant α-synuclein (D) or ubiquitin (E) in the substantia nigra after LPS injection. Scale bar: 50 µm.

One of the pathological hallmarks of PD is the formation of Lewy bodies, a proteinaceous cytoplasmic inclusion containing α-synuclein and ubiquitin [37]. Thus, we double immunostained midbrain sections with antibodies to TH and α-synuclein, or TH and ubiquitin to assess intracytoplasmic accumulations of these proteins following striatal inflammation. Fluorescent microscopic analysis of dual-stained TH and α-synuclein demonstrated smeared immunostaining of α-synuclein in the neuronal cytoplasm of the vehicle-injected side. However, an accumulation of α-synuclein in the cytoplasm of surviving TH-positive neurons was observed in the substantia nigra ipsilateral to the LPS injection (Fig. 3A,B). Immunofluorescent staining also revealed that ubiquitin accumulates in the spared dopaminergic neurons following intrastriatal LPS injections (Fig. 3C). In an attempt to visualize insoluble form of α-synuclein or ubiquitin, we pretreated midbrain sections with proteinase K, and then immunostained with antibodies against α-synuclein or ubiquitin. Intrastriatal LPS injection increased the immunoreactivity of α-synuclein (Fig. 3D) and ubiquitin (Fig. 3E) in the ipsilateral nigral neurons.

### 3. Behavioral impairments

**Figure 4:**
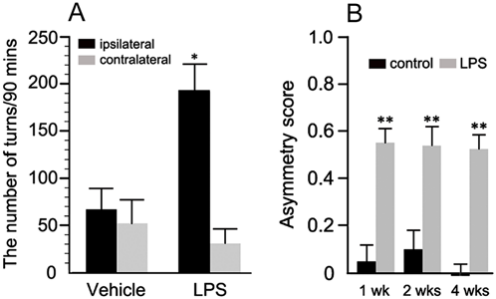
Behavioral deficits following intrastriatal LPS. (A) Ipsilateral rotational behavior in the unilateral LPS-injected animals is significantly increased relative to vehicle-treated animals when amphetamine was administered (n = 5–6/group; * p<0.05). (B) The cylinder test revealed that asymmetric forelimb use is increased significantly after intrastriatal LPS and was sustained for four weeks (n = 5–6/group; ** p<0.01).

Amphetamine-induced rotational behavior was analyzed to assess the unilateral degeneration of the presynaptic dopaminergic neuron terminals. Vehicle-injected rats did not show any significant bias in turning behavior after receiving an amphetamine injection. We did not observe the drug-induced rotational behavior at one week and two weeks after LPS challenge (data not shown). However, intrastriatal LPS caused a marked ipsilateral rotational behavior toward the lesioned side (p = 0.031) upon amphetamine challenge, four weeks after LPS injections (Fig. 4A).

The cylinder test was carried out at one week, two weeks, and four weeks post- LPS injection to assess an independent forelimb touch of animals to support their body against a cylinder wall [34]. Pronounced asymmetric forelimb use was developed by unilateral intrastriatal LPS injection, but not by vehicle injections. Asymmetric forelimb use was significantly increased at all three time points (p = 0.004, p = 0.005, and p = 0.013 respectively) (Fig. 4B).

### 4. Neuroinflammation in the nigrostriatal pathway after intrastriatal LPS injection

We characterized neuroinflammation by immunostaining of MHC class II (OX-6), a marker for activated microglia and by measuring the transcriptional induction of proinflammatory cytokine genes via the RNase protection assay following intrastriatal LPS injection. We also performed western blot analysis to see the temporal and regional patterns of iNOS expression after LPS challenge.

**Figure 5:**
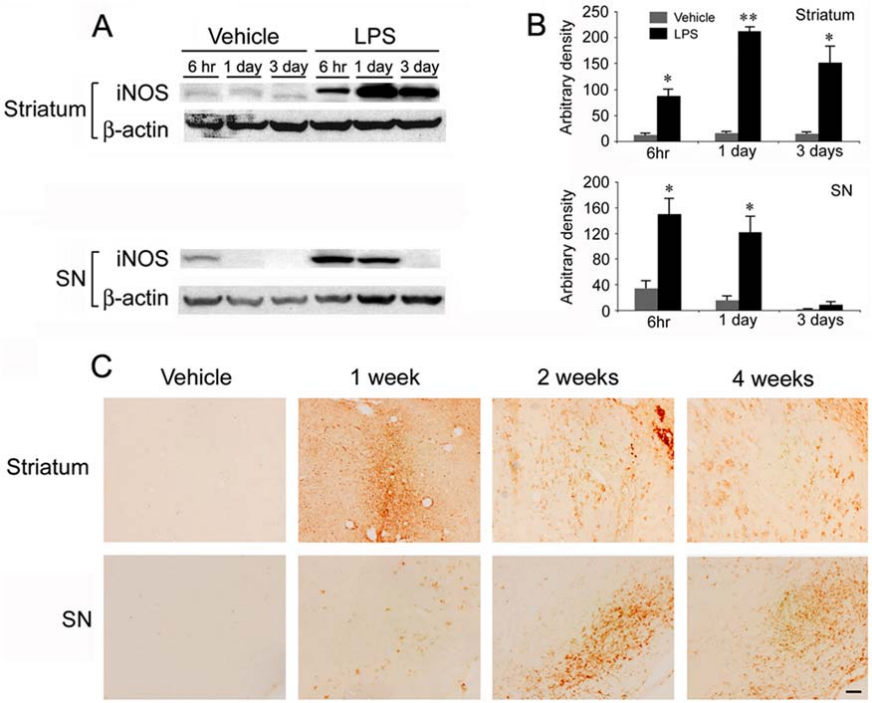
Microglial activation and elevation of iNOS expression in both the substantia nigra and striatum following intrastriatal LPS injection. The increased iNOS expression occurs at 6 hr post LPS injection, which is sustained for three days in the striatum, and one day in the substantia nigra (n = 3/group, * p<0.05, ** p<0.01 vs. control) (A,B). The OX-6 immunoreactivity in the LPS-injected striatum is markedly increased one week after LPS injections compared to control or the naïve side, and immunoreactivity of OX-6 gradually decreased over time. However, the immunoreactivity is still positive four weeks after LPS (C, top). OX-6-positive microglia appear in the substantia nigra one week after intrastriatal LPS injection, and the immunoreactivity peaks at two weeks and then decreases to some extent at four weeks (C, bottom). Scale bar: 50 µm.

Increased iNOS expression began to be detected 6 hr after LPS challenge in both the substantia nigra (p = 0.035) and striatum (p = 0.021) (Fig. 5A,B). The increased nigral iNOS immunoreactivity was gradually reduced as time passed, and returned to control level at three days (p = 0.042 at one day, and p = 0.265 at three days), while the increase in striatal iNOS expression reached a peak at one day and was still prominent at three days (p = 0.007 at one day, and p = 0.027 at three days) (Fig. 5A,B).

Injection of LPS into the striatum markedly increased the number of OX-6-positive microglia in the striatum at seven days post injections, and the increased immunoreactivity remained elevated for four weeks (Fig. 5C, top panel). Immunoreactivity for OX-6 began to appear in the ipsilateral substantia nigra one week after LPS injection, peaked at two weeks, and was still prominent four weeks after LPS (Fig. 5C, bottom panel).

Significant transcriptional increases of IL-1α (p = 0.006), TNF-α (p = 0.007), IL-1β (p = 0.003), and IL-6 (p = 0.001) were measured in the LPS-treated striatum three hours after LPS injection. The level of TNF-α mRNA remained significantly elevated for up to one day (p = 0.043) and IL-1β for three days (p = 0.027) after LPS (Fig. S3 a,b). Interestingly, there was a significant increase in the mRNA expression of IL-1β (p = 3×10^−4^) and IL-6 (p = 0.002) in the ipsilateral substantia nigra, as early as three hours (Fig. S3 c,d).

### 5. Mitochondrial dysfunction

**Figure 6:**
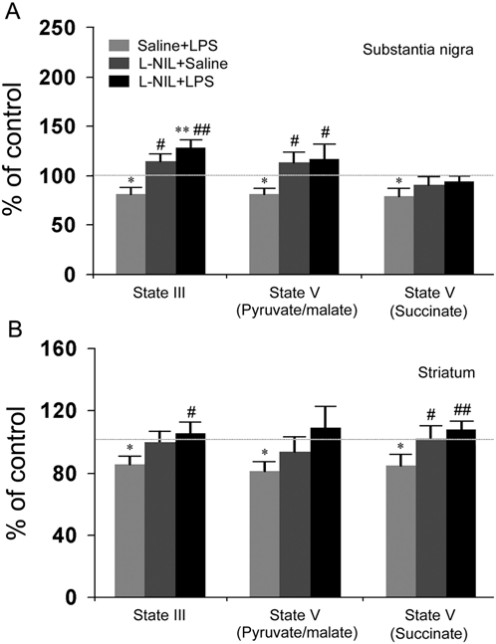
LPS impairs nigrostriatal mitochondria respiration. (A) Functional impairment occurs in the nigral mitochondria as LPS significantly decreases state III and state V respiration when driven by both complex I and complex II substrates. Treatment of L-NIL, an iNOS inhibitor prevents LPS-induced mitochondrial dysfunction (n = 6/group; * p<0.05, ** p<0.01 vs. saline+saline, # p<0.05, ## p<0.01 vs. saline+LPS). (B) It appears that there is a significant decrease in state III and state V respiration of striatal mitochondria when driven by both complex I and complex II substrates in the striatum ipsilateral to LPS injection. L-NIL efficiently blocks the neuroinflammation-mediated defect in striatal mitochondrial respiration (n = 9/group; * p<0.05, ** p<0.01 vs. Saline+Saline, # p<0.05, ## p<0.01 vs. Saline+LPS).

Intrastriatal LPS induced a significant decrease in nigral (81.3% of control, p = 0.045) and striatal (85.3% of control, p = 0.028) mitochondria state III respiration (the ability to phosphorylate ADP into ATP), which was efficiently prevented by treatment of L-NIL (Fig. 6A,B). When using the substrates pyruvate and malate, in the presence of carbonyl cyanide 4-trifluoromethoxy phenylhydrazone for maximum (state V) respiration, LPS induced a significant decrease in complex I activity of both nigral (80.9% of control, p = 0.035) and striatal (81.2% of control, p = 0.032) mitochondria. This impairment in complex I-driven state V respiration was blocked by treatment with the iNOS inhibitor. When utilizing succinate, the substrate for complex II driven respiration, state V respiration was also significantly reduced in the substantia nigra (79.0% of control, p = 0.021) and striatum (84.6% of control, p = 0.024) ipsilateral to LPS challenge (Fig. 6A,B). The reduction in complex II-driven state V respiration was prevented by treatment with L-NIL. This mitochondrial malfunction was not as marked as that of a previous study [30], which may reflect wash-off or severely damaged mitochondria during the isolation, as a Ficoll gradient method was used here.

### 6. S-nitrosylation and nitration of mitochondrial complex I

**Figure 7:**
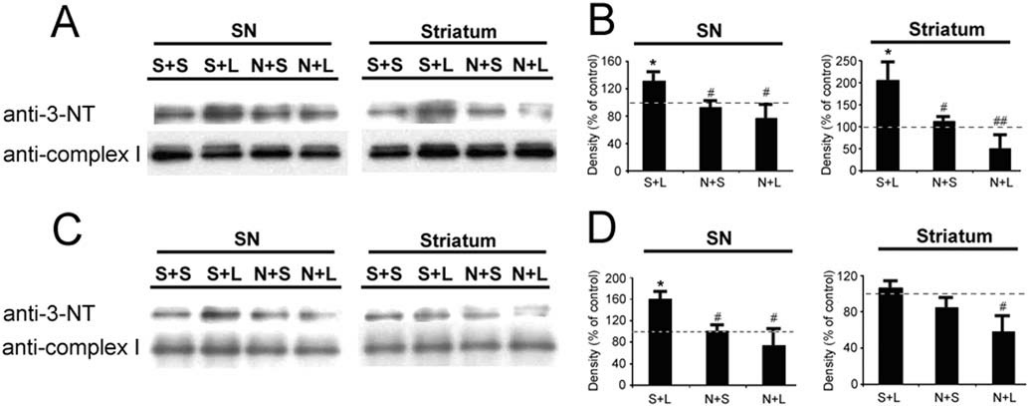
Nitration/S-nitrosylation of mitochondrial complex I after intrastriatal LPS. Intrastriatal LPS injection increases 3-nitrotyrosine (3-NT) level in complex I three days after LPS injection. Treatment with L-NIL appears to prevent the LPS-induced elevation of mitochondrial protein nitration (A,B). Isolated nigral and striatal mitochondria complex I proteins have an increase in S-nitrosylation three days after LPS injection. L-NIL treatment appears to prevent the LPS-induced increase of S-nitrosylation (C,D). S+S: saline treated and saline injected; S+L: saline-treated and LPS-injected; N+S: L-NIL-treated and saline-injected; and N+L: L-NIL-treated and LPS-injected; SN: substantia nigra (n = 4/group; * p<0.05 vs. S+S, and # p<0.05, p<0.01 vs. S+L).

To determine if S-nitrosylation or nitration of mitochondrial proteins is involved in the neuroinflammation-mediated mitochondrial dysfunction, we analyzed the nitration and/or S-nitrosylation level of mitochondrial complex I. Mitochondrial function can be compromised by extensive nitration or S-nitrosylation of mitochondrial complex I [18]. A significant elevated nitration of complex I (p = 0.038) occurred in the substantia nigra and striatum three days after LPS challenge (Fig. 7A,B). Treatment with L-NIL efficiently prevented the increased protein nitration. LPS injection significantly increased in S-nitrosylation of complex I in the substantia nigra, which was blocked by L-NIL injection (Fig. 7C,D).

## Discussion

Neuroinflammation, mitochondrial dysfunction and selective loss of dopaminergic neurons have been consistently observed in the substantia nigra of PD patients [1], [6], [20]. However, the correlation among these three in PD remains unclear. Here, we showed that neuroinflammation is able to mediate mitochondrial impairment by S-nitrosylation/nitration of mitochondrial proteins. This is followed by progressive dopaminergic neurodegeneration in the nigrostriatal system.

The loss of the dopaminergic neurons might be attributable to the intrinsic sensitivity of dopaminergic neurons to compromised mitochondrial function. Greenamyre and colleagues reported that systemic administration of a complex I inhibitor, rotenone to rats produced a selective degeneration of dopaminergic neurons in the substantia nigra [23]. Consistent with their report, we recently found that trichloroethylene causes selective loss of the nigral dopaminergic neurons in rats via complex I inhibition, and long term exposure to the chemical may be related to the development of parkinsonism in a group of factory workers [38]. Furthermore, mitochondrial dysfunction alone is sufficient to initiate parkinsonism in conditional knock-out mice by disrupting the gene for mitochondrial transcription factor A in the nigral dopamine neurons [39]. These observations are in agreement with the fact that rare familial forms of PD are related to mutations in the gene encoding PINK1 or DJ1, which both regulate mitochondrial function [40]. Thus, it can be questioned, what makes the nigral dopaminergic neurons substantially susceptible to mitochondrial dysfunction. A recent study showed that nigral dopamine neurons unusually rely on L-type voltage-gated calcium ion channels for basal activity, and the reliance increases with age [41]. The high dependency on the calcium channel leads to sustained elevation in cytosolic calcium concentration, which enhances mitochondrial respiration, reactive oxygen species generation, and ATP demand [42]–[44]. Therefore, the nigral dopaminergic neurons can be devastated by mitochondrial insults, which are tolerable to the other populations of neurons.

The implication of NO in PD pathogenesis is supported by the observations that iNOS expression is upregulated in activated microglia [11], [45], the immunoreactivity of nitrated α-synuclein is prominently positive in the Lewy bodies [46], [47], and several enzymes including parkin, peroxiredoxin-2, and protein-disulphide isomerase lose their function by S-nitrosylation in PD brains [48]–[50]. Although association of NO with mitochondrial dysfunction in PD is unclear, NO and its metabolite, peroxynitrite can compromise mitochondrial biomolecules to induce impairment in mitochondrial respiration. It has been well known that NO reversibly inhibit cytochrome c oxidase activity and depletes mitochondrial antioxidants such as glutathione [51]. Importantly, peroxynitrite and high level of NO irreversibly inactivates mitochondrial complex I activity by tyrosine nitration, S-nitrosylation, oxidation of residues and damage of iron sulfur center [18], [52]. These notions are in agreement with our results that intrastriatal LPS injection leads to complex I nitration and S-nitrosylation, and is accompanied decrease in mitochondrial respiration rate. This evidence strongly supports the hypothesis that excessive production of NO in the brain might contribute to mitochondrial dysfunction and subsequent neuronal energy deficiency observed in PD.

Inflammatory activation of microglia is related to increases in release of glutamate and consequent excitotoxicity [53]. It has been reported that neuronal NOS (nNOS)-derived NO accounts, at least in part, for glutamate-induced excitotoxicity [54]. We found that activated microglia appears one week after LPS challenge, and the microgliosis were sustained for four weeks in the substantia nigra. In addition, we observed that treatment of L-N(G)-nitro-arginine, an inhibitor of nNOS, was also neuroprotective against intrastriatal LPS in mice, suggesting the possible contribution of nNOS to the dopamine neurodegeneration [55]. This is further supported by our observation of cytoplasmic accumulation of α-synuclein and ubiquitin in the nigral dopamine-producing neurons, as extensive nitrosative/oxidative stress might cause impairment of the ubiquitin proteasome system resulting in accumulation of misfolded proteins [50], [56]. Support to the notion comes from the result that the accumulation of α-synuclein was attenuated by treatment with L-NIL (unpublished data). Hence, our results might be relevant for the molecular pathway for Lewy body formation and PD pathogenesis.

It is interesting that loss of a relatively small population of nigral dopamine neurons caused a decrease in the striatal dopamine level and behavioral impairment in the LPS-treated animals. The behavioral deficit began even at early time-point like one week following LPS injection. This phenomenon may suggest that the striatal inflammation antagonizes the function of the dopaminergic nigrostriatal pathway via altering synthesis and/or release of dopamine, or by modulating dopamine-mediated signal transduction before a significant demise of the dopaminergic neurons occurs. In support of this suggestion, it was shown that MPTP treatment induces striatal TH nitration, which was related to inactivation of the enzyme and a subsequent greater decline in dopamine level, compared to the loss of the dopaminergic neurons [57]. In addition, direct injection of proinflammatory mediators into the striatum such as prostaglandin D2 and the thromboxane A2 agonist induced impairment of motor behavior; although, the specific mechanism was not elucidated [58], [59]. Therefore, further study is required to fully illuminate the mechanism by which neurochemical alterations and behavioral deficit occur following intrastriatal LPS injections.

Taken together, results of the present study provide strong support to the hypothesis that neuroinflammation may significantly contribute to PD pathogenesis. This animal model may be useful for studying neuroinflammatory mechanisms by which nigral dopamine neurons degenerate in PD.

## Supporting Information

Figure S1 LPS-induced neuroinflammation significantly decreases TH-positive fiber density in the striatum four weeks after LPS challenge (n = 5/group, *** p<0.001 vs. control).
(2.50 MB TIF)

Figure S2 Decrease in striatal serotonin level following LPS injection. HPLC analysis reveals that intrastriatal LPS injection significantly reduces serotonin level, but not its metabolite 5-HIAA resulting in marked elevation of the turnover ratio (5-HIAA/serotonin) (n = 7/group, *** p<0.001 vs. control).
(2.59 MB TIF)

Figure S3 The elevated mRNA levels of proinflammatory cytokines in the striatum and substantia nigra. (A,B) RPA shows that the mRNA of the proinflammatory cytokines TNF-alpha, and IL-1beta begin to significantly increase three hours after LPS injections in the striatum, compared to the naïve or vehicle treated striatum, which is sustained for up to three days following LPS. The elevated mRNA levels of IL-1alpha and IL-6 occur three hours after LPS injection in the striatum, compared to the naïve or vehicle treated striatum, and return to control level at six hours following LPS (n = 4/group; * p<0.05, ** p<0.01). (C,D) RPA demonstrates that mRNA levels of IL-1beta and IL-6 are significantly increased in the substantia nigra three hours after LPS injection and remain significantly increased for six hours, when compared with the naïve or vehicle treated substantia nigra. IL-1alpah and TNF-alpha only show a trend to be increased at the three hour time point (n = 4/group; * p<0.05, ** p<0.01).
(5.90 MB TIF)
