# Supplementary figures and images for: Striatal Neuroinflammation Promotes Parkinsonism in Rats
Source: PLoS One. 2009 May 8;4(5):e5482. doi: 10.1371/journal.pone.0005482 (PMC2674956; doi:10.1371/journal.pone.0005482)

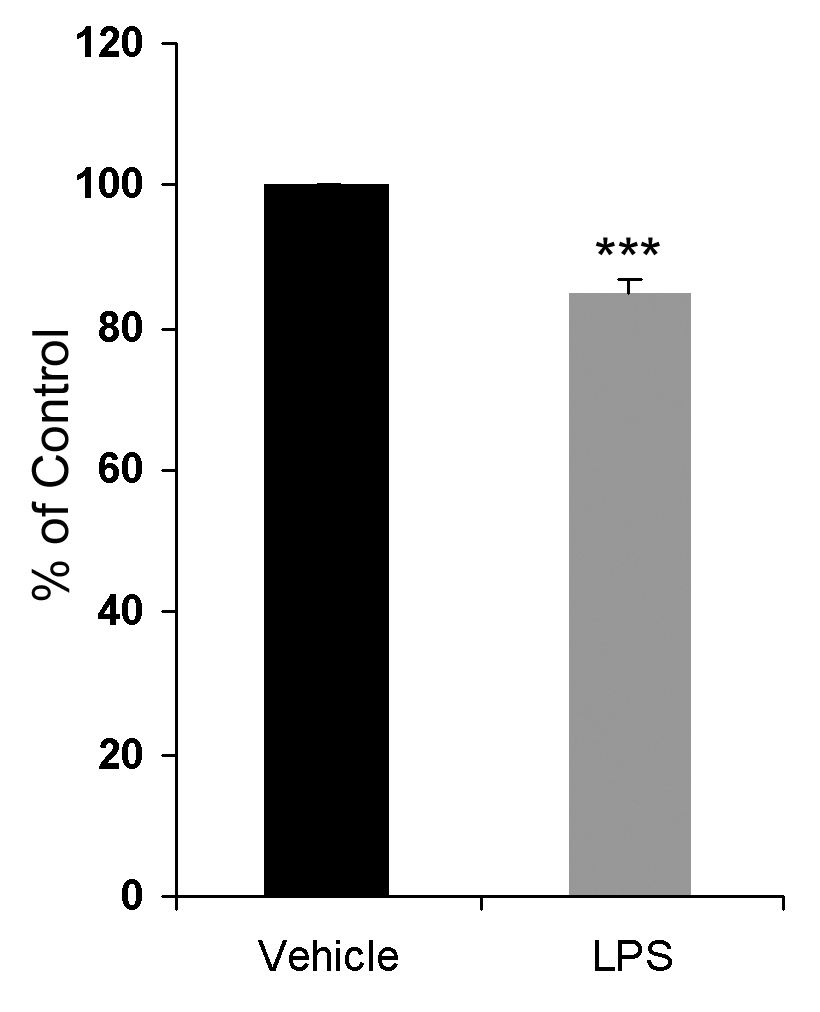

Supplement: Figure S1 — LPS-induced neuroinflammation significantly decreases TH-positive fiber density in the striatum four weeks after LPS challenge (n = 5/group, *** p<0.001 vs. control). (2.50 MB TIF) [file pone.0005482.s001.tif]

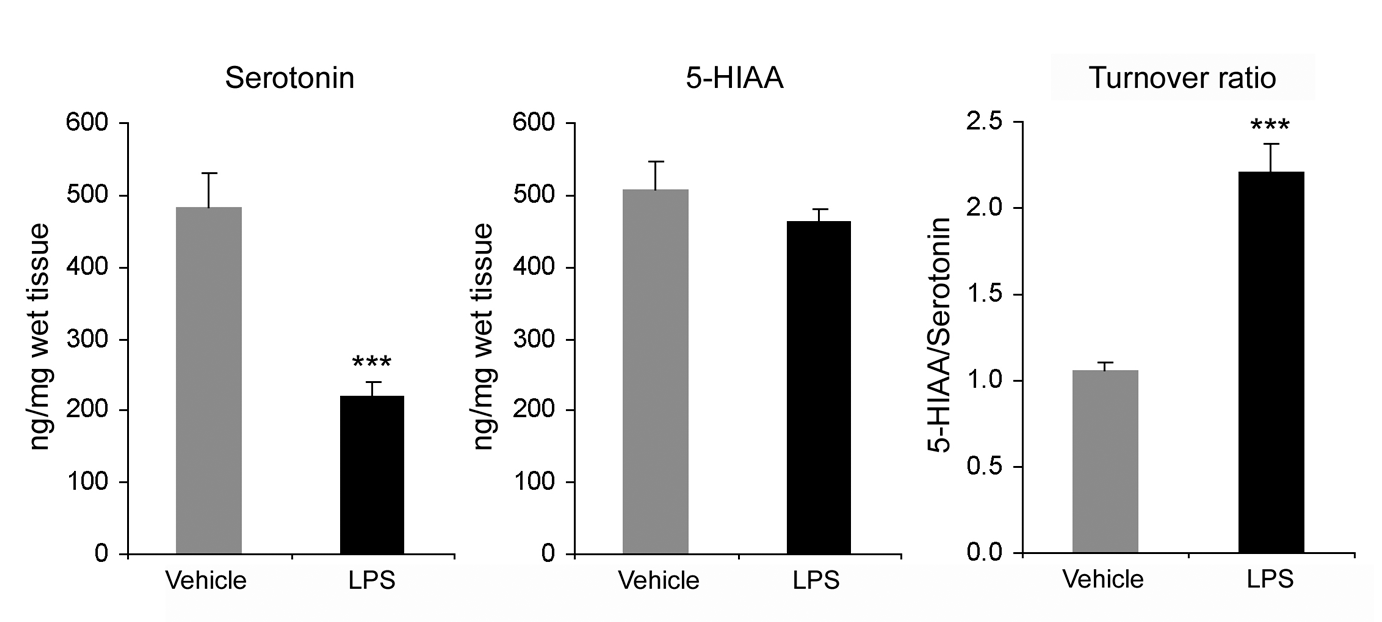

Supplement: Figure S2 — Decrease in striatal serotonin level following LPS injection. HPLC analysis reveals that intrastriatal LPS injection significantly reduces serotonin level, but not its metabolite 5-HIAA resulting in marked elevation of the turnover ratio (5-HIAA/serotonin) (n = 7/group, *** p<0.001 vs. control). (2.59 MB TIF) [file pone.0005482.s002.tif]

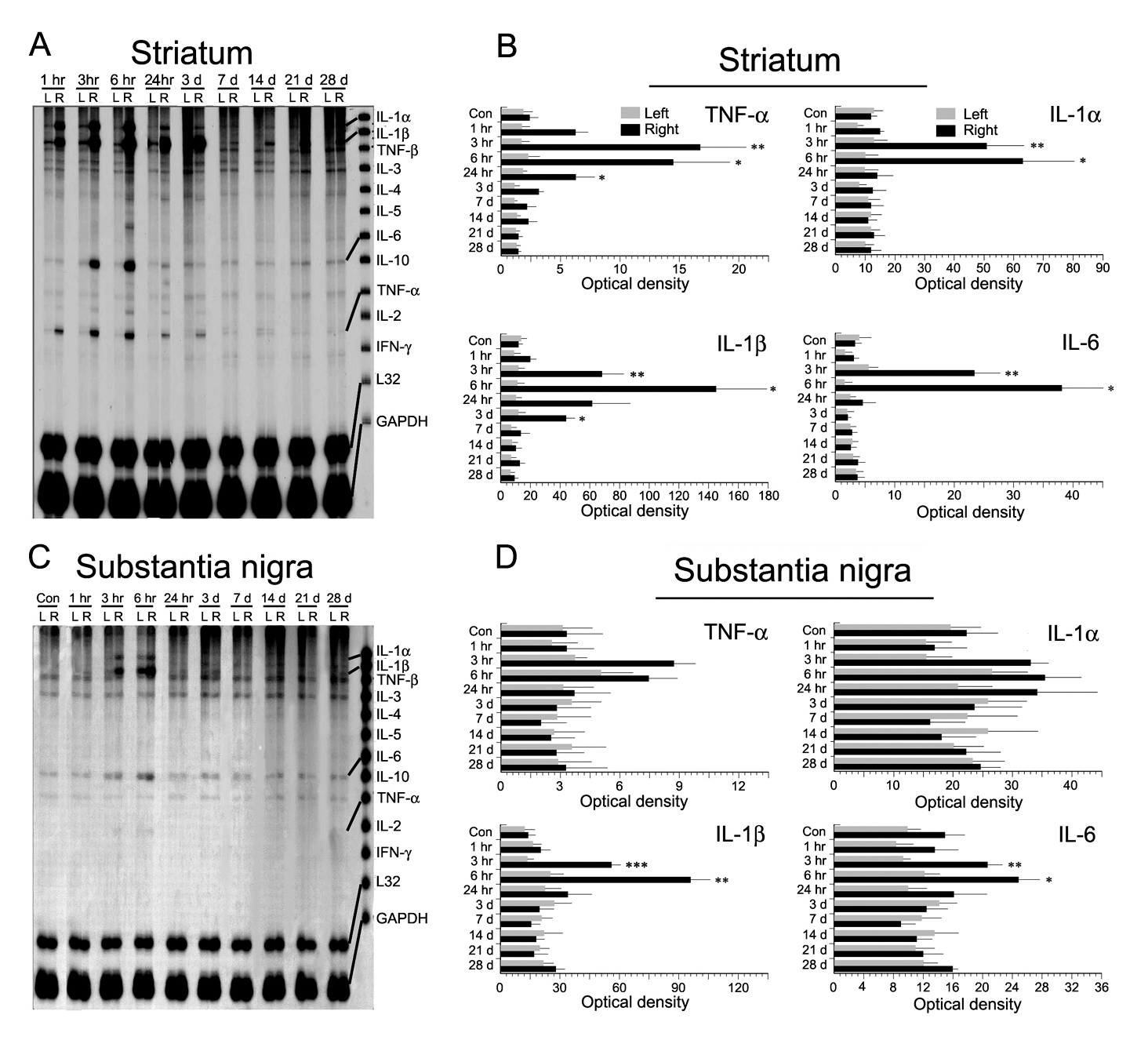

Supplement: Figure S3 — The elevated mRNA levels of proinflammatory cytokines in the striatum and substantia nigra. (A,B) RPA shows that the mRNA of the proinflammatory cytokines TNF-alpha, and IL-1beta begin to significantly increase three hours after LPS injections in the striatum, compared to the naïve or vehicle treated striatum, which is sustained for up to three days following LPS. The elevated mRNA levels of IL-1alpha and IL-6 occur three hours after LPS injection in the striatum, compared to the naïve or vehicle treated striatum, and return to control level at six hours following LPS (n = 4/group; * p<0.05, ** p<0.01). (C,D) RPA demonstrates that mRNA levels of IL-1beta and IL-6 are significantly increased in the substantia nigra three hours after LPS injection and remain significantly increased for six hours, when compared with the naïve or vehicle treated substantia nigra. IL-1alpah and TNF-alpha only show a trend to be increased at the three hour time point (n = 4/group; * p<0.05, ** p<0.01). (5.90 MB TIF) [file pone.0005482.s003.tif]
